# Supplementary material for: Impact of circ-0000221 in the Pathogenesis of Hepatocellular via Modulation of miR-661–PTPN11 mRNA Axis
Source: Pharmaceutics. 2022 Jan 6;14(1):138. doi: 10.3390/pharmaceutics14010138 (PMC8778063; doi:10.3390/pharmaceutics14010138)

pharmaceutics

MDPI

Supplementary Materials: Impact of circ-0000221 in the Pathogenesis of Hepatocellular via Modulation of miR-661–PTPN11 mRNA Axis

Marwa Matboli, Mohmed K. Hassan, Mahmoud A. Ali, Mohamed Tarek Mansour, Waheba Elsayed, Reham Atteya, Hebatallah Said Aly, Mahmoud El Meteini, Hesham Elghazaly, Sherif El-Khamisy and Sara H.A. Agwa

Figure S1. Print screen Shot fot hsa_circ_000022 predicted by Circ 2 trait : http://gyanxet-beta.com/circdb/, accessed on 10. November 2021.

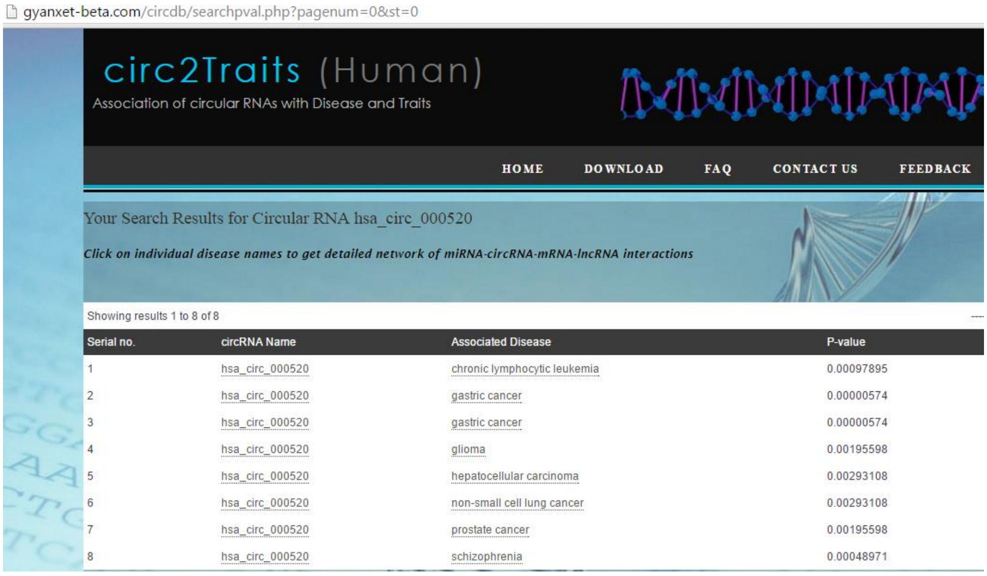

Figure S2. Print screen Shot fot hsa_circ_000022 & miR-661 predicted by Circ 2 trait : http://gyanxet-beta.com/circdb/, accessed on 10. November 2021.

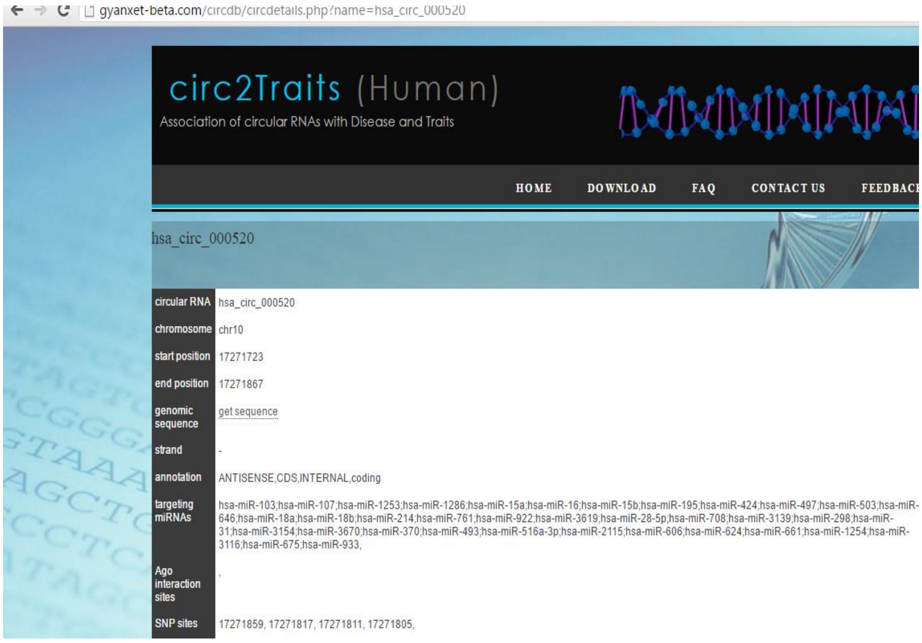

Figure S3.: Print screen Shot fot miR-661& PTPN-11 predicted by Target scan; Available at http://amp.pharm.mssm.edu/Harmonizome/gene_set/hsa-miR-661/TargetScan+Predicted+Conserved+microRNA+Targets accessed on 10. November 2021.

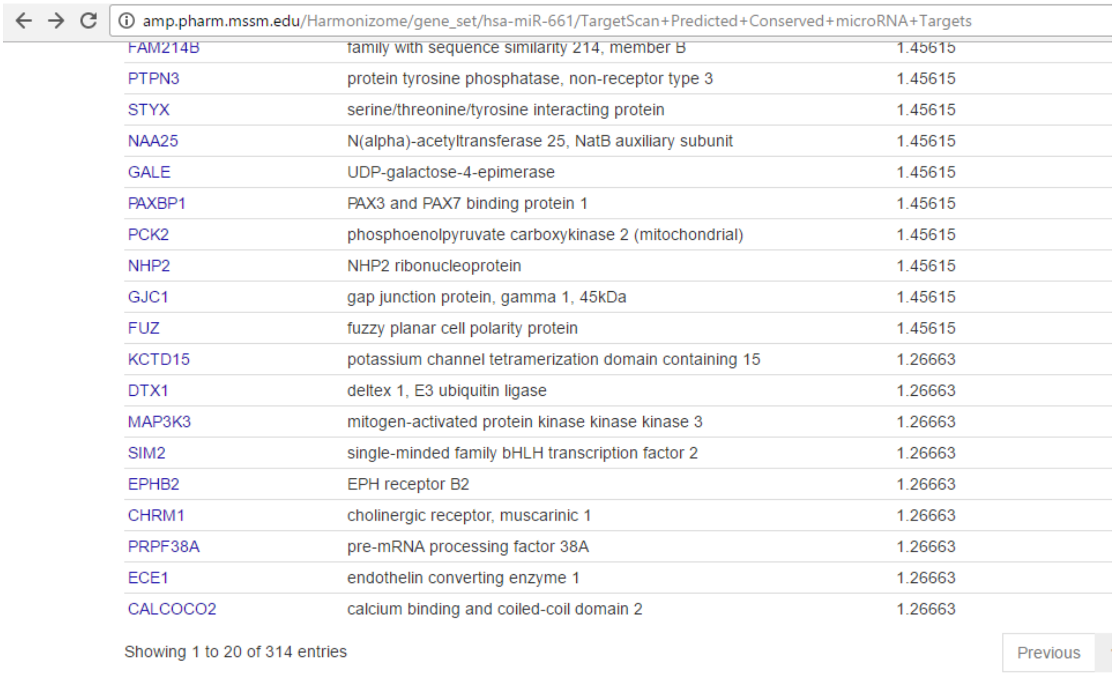

amp.pharm.mssm.edu/Harmonizome/gene_set/hsa-miR-661/TargetScan+Predicted+Conserved+microRNA+Targets

FAM214B	family with sequence similarity 214, member B	1.45615
PTPN3	protein tyrosine phosphatase, non-receptor type 3	1.45615
STYX	serine/threonine/tyrosine interacting protein	1.45615
NAA25	N(alpha)-acetyltransferase 25, NatB auxiliary subunit	1.45615
GALE	UDP-galactose-4-epimerase	1.45615
PAXBP1	PAX3 and PAX7 binding protein 1	1.45615
PCK2	phosphoenolpyruvate carboxykinase 2 (mitochondrial)	1.45615
NHP2	NHP2 ribonucleoprotein	1.45615
GJC1	gap junction protein, gamma 1, 45kDa	1.45615
FUZ	fuzzy planar cell polarity protein	1.45615
KCTD15	potassium channel tetramerization domain containing 15	1.26663
DTX1	deltex 1, E3 ubiquitin ligase	1.26663
MAP3K3	mitogen-activated protein kinase kinase kinase 3	1.26663
SIM2	single-minded family bHLH transcription factor 2	1.26663
EPHB2	EPH receptor B2	1.26663
CHRM1	cholinergic receptor, muscarinic 1	1.26663
PRPF38A	pre-mRNA processing factor 38A	1.26663
ECE1	endothelin converting enzyme 1	1.26663
CALCOCO2	calcium binding and coiled-coil domain 2	1.26663

Showing 1 to 20 of 314 entries

Previous

Figure S4. Exogenous expression of hsa_circ_000022 significantly reduced the colony-forming ability of the SNU449 and HepG2 cells.

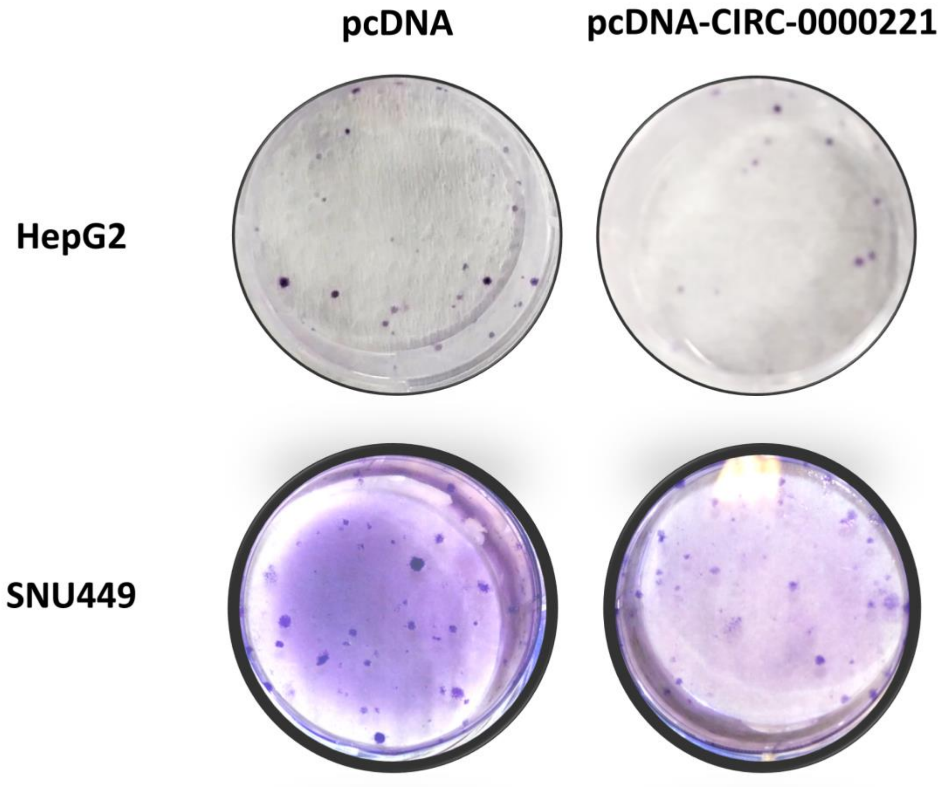

Supplement: Supplementary file 1 [file pharmaceutics-14-00138-s001.zip › pharmaceutics-1501330-supplementary.pdf]
